# Gut Microbiota Profiling and Gut–Brain Crosstalk in Children Affected by Pediatric Acute-Onset Neuropsychiatric Syndrome and Pediatric Autoimmune Neuropsychiatric Disorders Associated With Streptococcal Infections

**DOI:** 10.3389/fmicb.2018.00675

**Published:** 2018-04-06

**Authors:** Andrea Quagliariello, Federica Del Chierico, Alessandra Russo, Sofia Reddel, Giulia Conte, Loris R. Lopetuso, Gianluca Ianiro, Bruno Dallapiccola, Francesco Cardona, Antonio Gasbarrini, Lorenza Putignani

**Affiliations:** ^1^Unit of Human Microbiome, Bambino Gesù Children’s Hospital, IRCCS, Rome, Italy; ^2^Department of Human Neurosciences, Sapienza University of Rome, Rome, Italy; ^3^Department of Internal Medicine, Gastroenterology and Hepatology, Catholic University of the Sacred Heart, Agostino Gemelli Hospital, Rome, Italy; ^4^Scientific Directorate, Bambino Gesù Children’s Hospital, IRCCS, Rome, Italy; ^5^Unit of Parasitology Bambino Gesù Children’s Hospital, IRCCS, Rome, Italy

**Keywords:** PANS, PANDAS, gut microbiota, dysbiosis, bacterial biomarkers

## Abstract

Pediatric acute-onset neuropsychiatric syndrome (PANS) and pediatric autoimmune neuropsychiatric disorders associated with streptococcal infections syndrome (PANDAS) are conditions that impair brain normal neurologic function, resulting in the sudden onset of tics, obsessive-compulsive disorder, and other behavioral symptoms. Recent studies have emphasized the crosstalk between gut and brain, highlighting how gut composition can influence behavior and brain functions. Thus, the present study investigates the relationship between PANS/PANDAS and gut microbiota ecology. The gut composition of a cohort of 30 patients with PANS/PANDAS was analyzed and compared to control subjects using 16S rRNA-based metagenomics. Data were analyzed for their α- and β-diversity; differences in bacterial distribution were detected by Wilcoxon and LEfSe tests, while metabolic profile was predicted via PICRUSt software. These analyses demonstrate the presence of an altered bacterial community structure in PANS/PANDAS patients with respect to controls. In particular, ecological analysis revealed the presence of two main clusters of subjects based on age range. Thus, to avoid age bias, data from patients and controls were split into two groups: 4–8 years old and >9 years old. The younger PANS/PANDAS group was characterized by a strong increase in Bacteroidetes; in particular, *Bacteroides*, *Odoribacter*, and *Oscillospira* were identified as potential microbial biomarkers of this composition type. Moreover, this group exhibited an increase of several pathways concerning the modulation of the antibody response to inflammation within the gut as well as a decrease in pathways involved in brain function (i.e., SCFA, D-alanine and tyrosine metabolism, and the dopamine pathway). The older group of patients displayed a less uniform bacterial profile, thus impairing the identification of distinct biomarkers. Finally, Pearson’s analysis between bacteria and anti-streptolysin O titer reveled a negative correlation between genera belonging to Firmicutes phylum and anti-streptolysin O while a positive correlation was observed with *Odoribacter*. In conclusion, this study suggests that streptococcal infections alter gut bacterial communities leading to a pro-inflammatory status through the selection of specific bacterial strains associated with gut inflammation and immune response activation. These findings highlight the possibility of studying bacterial biomarkers associated with this disorder and might led to novel potential therapeutic strategies.

## Introduction

Pediatric acute-onset neuropsychiatric syndrome (PANS) refers to a clinical spectrum of neuropsychiatric disorders triggered by environmental conditions, metabolic disorders, and/or infections (Swedo et al., 2012). Children with PANS experience a striking onset of neuropsychiatric symptoms, which include obsessions/compulsions, food restriction, personality changes, emotional lability, sleep disturbances, and even characteristics of mood disorders (Mangiola et al., 2016). Subjects are usually initially diagnosed with obsessive-compulsive disorder (OCD) and/or an eating disorder, but the sudden onset of symptoms distinguishes PANS from the other similar conditions.

The concept of PANS is relatively recent and is derived from research on pediatric autoimmune neuropsychiatric disorders associated with streptococcal infections syndrome (PANDAS); PANDAS is now considered as a specific subset within the broader clinical spectrum of PANS.

PANDAS was first described in 1998 by Dr. Swedo of the National Institute of Mental Health (NIMH), who proposed five diagnostic criteria: (1) the presence of tic and/or OCDs; (2) prepubertal onset; (3) acute symptom onset and relapsing-remitting course; (4) temporal association with Group A streptococcal infection (GAS); and (5) the presence of associated neurologic abnormalities (Swedo et al., 1998). Additional symptoms observed in these patients were loss of motor skills, behavioral disorders (i.e., hyperactivity, mood changes, and irritability), and food restriction (Murphy et al., 2004).

The temporal association with GAS infection led to the hypothesis of an autoimmune pathogenesis of this disorder similar to Sydenham chorea, an established sequela of rheumatic fever in which specific streptococcal antibodies cross-react against some brain antigens through the process of molecular mimicry (Murphy et al., 2010; Frankovich et al., 2015). Although some studies (Kirvan et al., 2006; Toufexis et al., 2015) have demonstrated clinical homogeneity of PANS/PANDAS patients, certain aspects of this condition still remain elusive (i.e., the interval between the inciting infection and the onset of symptoms), leading to a heated scientific debate (Macerollo and Martino, 2014). Data reported thus far have failed to identify a distinct biological marker for PANS/PANDAS. Recently, there has been a growing interest in gut microbiota (GM), particularly regarding its relationship with brain function (the so-called gut–brain axis; Clapp et al., 2017). Current advances in sequencing and bioinformatics technologies have allowed the investigation of possible correlations between gut microbial composition and several pathological conditions including inflammatory bowel disease (IBD), metabolic disorders, allergies, and neurological disorders (Karlsson et al., 2013; Ferreira et al., 2014; Putignani et al., 2016; Rachid and Chatila, 2016; Dinan and Cryan, 2017; Dzidic et al., 2017). A bidirectional communication appears to exist between the gut and the brain, through which each can influence the other. In particular, the gut interacts with the brain through the spinal cord, the enteric nervous system, the hypothalamic pituitary adrenal axis, and the central nervous system (CNS; Carabotti et al., 2015). This relationship is well supported by studies that have investigated the effects of probiotics, antibiotics, or even germ-free animals on brain activity and function (Desbonnet et al., 2015; Diaz Heijtz, 2016; Hoban et al., 2016). Given these data, the present work seeks to assess the composition of GM in subjects affected by PANS/PANDAS in order to obtain further physiological information regarding the gut–brain axis in this poorly understood disorder. The identification of gut microbial biomarkers of disease could play an important role in PANS/PANDAS clinical management.

## Materials and Methods

### Patients

A total of 30 consecutive PANS/PANDAS patients aged 4–16 years (average age 10.03 years, SD ± 3.47; 20 males and 10 females) were recruited at the Umberto I Hospital (Rome, Italy) and Agostino Gemelli Hospital (Rome, Italy) and sent to the Unit of Human Microbiome of “Bambino Gesù” Children’s Hospital (OPBG; Rome, Italy) for microbial diagnostics procedures.

Diagnosis of PANS/PANDAS was made based on the current diagnostic criteria (Swedo et al., 2004; Chang et al., 2015). From each patient, the following data were collected: age, gender, the presence of gastrointestinal disorders, the presence of inflammatory and/or infective and/or chronic diseases, asthma, allergies, consumption of antibiotics and probiotics, diet, and anti-streptolysin O titer (ASLOT; **Table 1**). Patients were age-matched with a cohort of 70 healthy controls (CTRL), screened using a survey provided by the OPBG Human Microbiome Unit on pediatric GM programming.

**Table 1:** Anamnestic and clinical data collected for each patient.

Sample ID^1^	Group	Age range	Gender	Gastrointestinal disturbs	Inflammatory disease	Infective disease	Chronic disease	Asthma	Allergies	Antibiotics^2^	Probiotics^3^	Diet	ASLOT (IU/ml)^4^
GUT_260	o-PAN	11–12	F	No	No	No	No	No	No	Penicillin	No	Omnivore	1311^∗^
GUT_268		11–12	M	Reflux	No	No	No	Yes	Dermatophagoides	No	Yes	Omnivore	1063^∗^
GUT_307		11–12	M	Diarrhea, abdominal pain	No	No	No	No	No	Penicillin	Yes	Omnivore	NA
GUT_312		11–12	F	No	No	No	No	No	No	No	No	Omnivore	350
GUT_379		11–12	M	Abdominal pain	No	No	No	No	No	Penicillin	No	Omnivore	89
GUT_249		13–16	M	Abdominal pain	No	No	No	No	Dermatophagoides	Penicillin	No	Omnivore	214
GUT_270		13–16	F	Constipation	No	No	No	No	Kiwi	No	No	Omnivore	40
GUT_539		13–16	M	No	No	No	No	No	No	No	No	Omnivore	150
GUT_559		13–16	M	Reflux	No	No	No	No	No	Penicillin	No	Omnivore	NA
GUT_585		13–16	F	Constipation	No	No	No	No	Gluten	No	Yes	Omnivore	180
GUT_593		13–16	F	No	No	No	No	No	No	No	No	Omnivore	459
GUT_345		20–30	F	Abdominal pain	No	No	No	No	No	No	No	Omnivore	877^∗^
GUT_186		9–10	F	Diarrhea	No	No	No	No	Grass pollen	Azithromycin	Yes	Omnivore	400
GUT_192		9–10	M	Abdominal pain	No	No	No	No	No	No	No	Omnivore	185
GUT_229		9–10	F	No	No	No	No	No	No	No	No	Omnivore	564^∗^
GUT_23		9–10	M	No	No	No	No	No	No	No	No	Omnivore	150
GUT_358		9–10	M	No	No	No	No	No	Yes	No	No	Omnivore	113
GUT_498		9–10	M	Diarrhea	No	No	No	Yes	Grass pollen	Azithromycin	Yes	Omnivore	245
GUT_53		9–10	M	No	No	No	No	No	No	No	No	Omnivore	NA
GUT_530		9–10	M	Vomit	No	No	No	No	No	Azithromycin	Yes	Omnivore	999^∗^
GUT_615		9–10	M	No	No	No	No	No	No	No	No	Omnivore	341
GUT_638		9–10	M	No	No	No	No	No	No	Penicillin	No	Omnivore	NA
GUT_785		9–10	M	Constipation	No	No	No	No	No	No	Yes	Omnivore	NA
GUT_786		9–10	M	No	No	No	No	No	Albumen	Penicillin	No	Omnivore	NA
GUT_357	y-PAN	4–6	M	No	No	No	No	No	No	No	No	Omnivore	922^∗^
GUT_180		7–8	F	No	No	No	No	No	No	No	Yes	Omnivore	24
GUT_200		7–8	M	Diarrhea	No	No	No	No	No	No	No	Omnivore	997^∗^
GUT_263		7–8	M	Constipation	No	No	No	No	No	No	Yes	Gluten-free, omnivore	255
GUT_274		7–8	F	No	No	No	No	No	No	No	No	Omnivore	965^∗^
GUT_576		7–8	M	No	No	No	No	No	No	No	No	Omnivore	316


*^*1*^Patient code; ^*2*^antibiotic intake was suspended from 4 to 2 months before GM test; ^*3*^probiotics intake was suspended from 4 to 2 months before GM test; ^*4*^anti-streptolysin O titer; asterisk “^∗^” indicates samples included in Pearson’s correlation analysis.*

Inclusion criteria for both PANS/PANDAS patients and CTRL were the absence of any inflammatory, infective, and/or chronic diseases at the time of the microbiota analysis; all antibiotic and pre-probiotic intake was to have been suspended beginning 4 or 2 months prior to the GM test (**Table 1**).

The study protocols (1404_OPBG_2017) were approved by the OPBG Ethics Research Committee, in accordance with the Declaration of Helsinki (as revised in Seoul, South Korea, October 2008).

### DNA Extraction and Targeted Metagenomics

All fecal samples collected from PANS/PANDAS patients and CTRL subjects were handled at the OPBG Human Microbiome Unit for biobanking and targeted-metagenomics processing. For each patient, three stool samples from consecutive days were collected and genomic DNA was extracted following the QIAamp DNA Stool Mini Kit (Qiagen, Germany) procedure. Next, the bacterial 16S ribosomal RNA (rRNA) gene was amplified using the primer set specific for the V1–V3 regions and the obtained PCR products were purified, quantified, and pooled in an equimolar way in a final amplicon library that was finally sequenced on a 454-Junior Genome Sequencer (Roche 454 Life Sciences, Branford, FL, United States). A first screening analysis of read counts versus α-diversity indices (i.e., Observed) was used to avoid stochastic effects during PCR and sequencing processes, thereby selecting the sample with the highest values of reads and α-diversity indices out of three collected samples to represent the microbial variability present in the gut of each subject.

### MG Data Open Access Repository

All 454 sequencing raw reads and associated metadata are available at NCBI: Bioprojects: PRJNA420009, gut metagenomic profile from PANS/PANDAS patients; PRJNA280490, gut metagenomic profile from healthy subjects^1^.

### Data Analysis

Reads were analyzed using the micca v1.6 pipeline^2^ (Albanese et al., 2015). To characterize the taxonomic structure of the samples, sequences were organized into operational taxonomic units (OTUs) by clustering at a threshold of 97% pairwise identity; representative sequences were classified using the VSEARCH-based consensus classifier and the Greenenes 13_8 database (Rognes et al., 2016). Then, the PyNAST v.0.1 program was used to perform a multiple sequence alignment (MSA; Caporaso et al., 2010) against the Greengenes 13_08 database filtered at 97% similarity for bacterial sequences; the MSA was then used to build the phylogenetic tree (DeSantis et al., 2006).

Statistical analyses were computed using the R package, *phyloseq*, for α- and β-diversity (McMurdie and Holmes, 2013), while the identification of differences in the relative abundances of taxa as well as the OTU correlation between PANS/PANDAS patients and CTRL subjects was assigned using Wilcoxon rank-sum (corrected for FDR) and Pearson tests (*Hmisc* package in R), respectively. Furthermore, the adonis function in the R package, *vegan*, was used to perform a PERMANOVA test on β-diversity with 999 permutations considering even age groups (corresponding to 4–6, 7–8, 9–10, 11–12, 13–16, and 20–30 years old) using the “*strata*” argument within the *adonis* function.

To gain more insight into the metagenomic function of the PANS/PANDAS patients microbiome, the PICRUSt v.1.1.0 tool was used (Langille et al., 2013); function predictions were analyzed using the HUMAnN2 v0.99 program to identify the Kyoto Encyclopedia of Genes and Genomes (KEGGs) pathway^3^ (Abubucker et al., 2012).

Finally, to identify possible OTU and KEGG biomarkers associated with PANS/PANDAS, a linear discriminant effect size (LEfSe) analysis was performed (Segata et al., 2011) with α value for the statistical test equal to 0.05 and a logarithmic LDA score threshold of 2.0.

## Results

### Bacterial Ecology and Distribution in PANS/PANDAS and CTRL Groups

**FIGURE 1:**
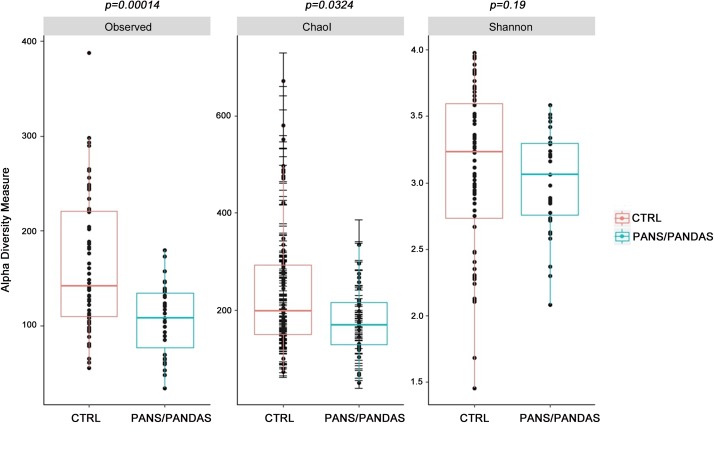
Boxplots representing α-diversity indices: Observed, Chao1, and Shannon. The plots represent the median, 25th, and 75th percentiles calculated for PANS/PANDAS (blue) and CTRL (red) groups. The corresponding *p*-values are reported on the top of each index.

After performing quality assessment and filtering of the reads, the read counts and Observed values for each of the three samples provided by each patient were evaluated in order to select the most representative sample for each subject (**Supplementary Figure S1**). Then, the GM biodiversity for both PANS/PANDAS and CTRL groups was analyzed via α- and β-diversity values. PANS/PANDAS patients showed an overall lower level of all α-biodiversity indices taken into account in this study (**Figure 1**), which was statistically significant using the Wilcoxon test for Observed (*p* < 0.01) and Chao1 indices (*p* < 0.05), while statistical significance was not reached for the Shannon index, although PANS/PANDAS subjects exhibited lower mean values.

**FIGURE 2:**
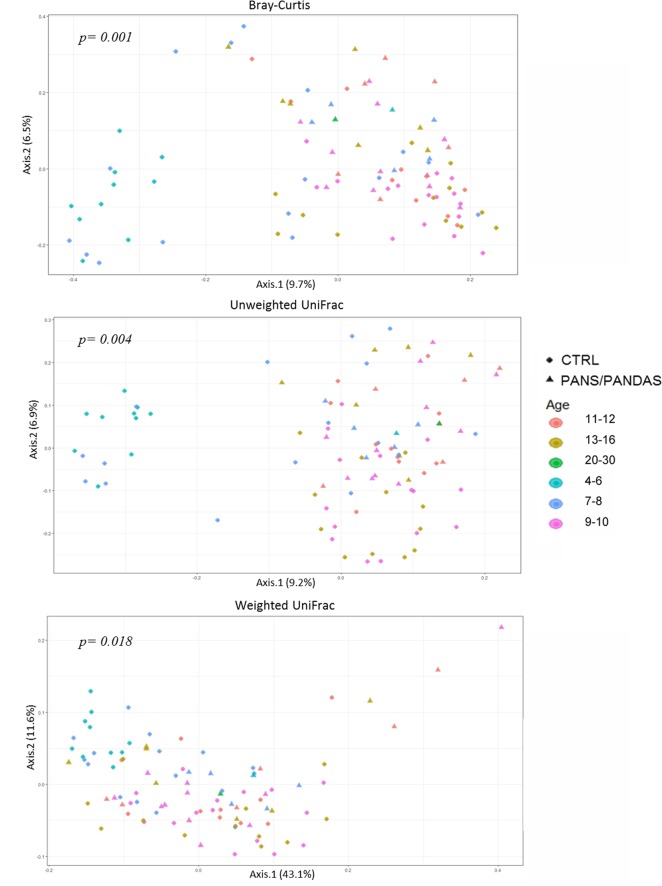
Principal coordinate analysis plot of PANS/PANDAS and CTRL groups. The plots show the first two principal coordinates (axes) for principal coordinates analysis (PCoA) using Bray–Curtis **(A)**, unweighted UniFrac **(B)**, and weighted UniFrac **(C)** algorithms. The resulting *p*-values for PERMANOVA analyses are reported in the figures.

Three ecological indices were used to evaluate the compositional dissimilarity between groups both in terms of species abundance (i.e., Bray–Curtis distance) and incorporating their phylogenetic relatedness (i.e., unweighted and weighted UniFrac). When analyzing β-diversity, a strong separation on the first axis (PERMANOVA *p* < 0.01) was observed for both Bray–Curtis and unweighted UniFrac analyses in which a small subset of CTRL samples was substantially separated from the rest of the dataset (**Figure 2**). Plotting the data by age range, the separated samples were found to be restricted to subjects in the 4–6 and 7–8 years old age CTRL groups.

Though less evident, a similar trend was visible (PERMANOVA *p* < 0.05) even when utilizing the weighted UniFrac analysis. Therefore, in order to prevent possible bias linked to age-related patterns of variability rather than to the disorder, subsequent analyses were carried out by dividing the global dataset into two age classes: a first group including subjects in the 4–8 year range (y-PAN) and a second group including subjects >9 years old (o-PAN). CTRL subjects were divided into the same age classes and matched with their peers.

**FIGURE 3:**
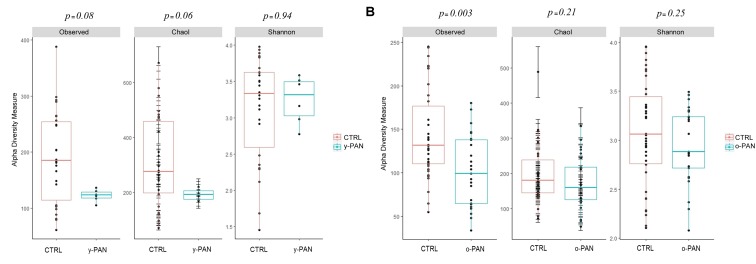
Boxplots representing α-diversity indices: Observed, Chao1, and Shannon. The plots represent the median, 25th, and 75th percentiles calculated for PANS/PANDAS (blue) and CTRL (red) group. Here, subjects (both patients and CTRL) are first segregated by age range: **(A)** y-PAN (4–8 years old) and **(B)** o-PAN (>9 years old). The corresponding *p*-values are reported on the top of each index.

The α-diversity analysis recomputed using the y-PAN group confirmed the previously observed profile. In particular, y-PAN patients exhibited even lower values of α-diversity indices (**Figure 3A**). Although lacking statistical significance, y-PAN patients also exhibited a trend toward reduced biodiversity and a high homogeneity for both the Observed and Chao1 indices, while they placed near to the CTRL group for the Shannon index. These differences are reflected in the distribution of relative abundances of bacterial families and genera among the three groups (**Supplementary Figure S2**).

### Microbiota Description and Metabolic Profile Prediction in y-PAN and CTRL

**FIGURE 4:**
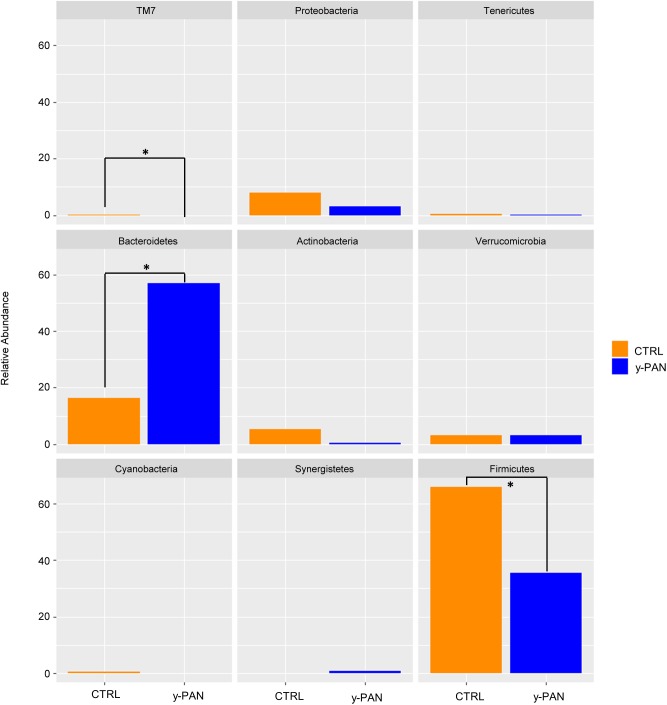
Bar chart representing Wilcoxon test results on operational taxonomic units (OTUs) at the phylum level of gut microbiota from the CTRL and y-PAN groups. Error bars for y-PAN patients are reported in blue while they are in orange for the CTRL subjects. The asterisks indicate the statistical significance (*p* < 0.05) between the two groups.

To look for distinctive features of the y-PAN group, taxa distribution was investigated at the phyla, family, and genus levels. Results of the Wilcoxon test highlighted OTU abundance differences at the phylum level (**Figure 4**), with a higher percentage of Bacteroidetes and a lower level of Firmicutes in y-PAN children compared to CTRL subjects. In addition, subjects in the y-PAN group exhibited a very low although non-significant distribution of Actinobacteria and a total absence of the TM7 phylum.

**FIGURE 5:**
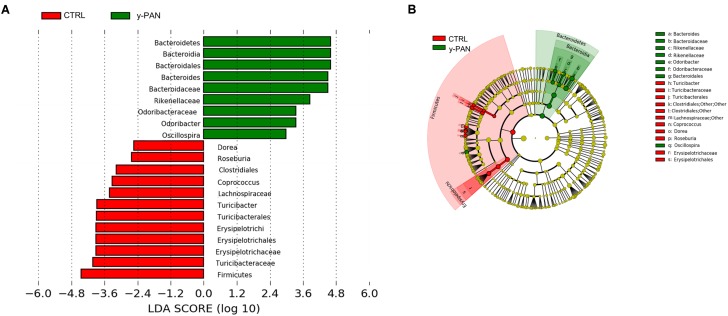
OTU biomarkers associated with y-PAN and CTRL groups. **(A)** A linear discriminant effect size (LeFse) analysis was performed (α value = 0.05, logarithmic LDA score threshold = 2.0). **(B)** The cladogram represents the phylogenetic relationship of significant OTUs associated with each group.

At the family level, most members of the Bacteroidetes phylum had higher relative abundance within y-PAN group, particularly Bacteroidaceae (with a mean value higher than 50%), Rikenellaceae, and Odoribacteriaceae. Conversely, several Firmicutes families such as Turicibacteraceae, Tissierellaceae, Gemellaceae, and Carnobacteriaceae (of the Bacilli class); Corynebacteriaceae and Lachnospiraceae (of the Clostridia class); and Erysipelotrichaceae were absent. Even Bifidobacteriaceae (Actinobacteria) were completely missing from the GM of y-PAN patients in this young age class (**Supplementary Figure S3**). Based on the OTU distribution, the LEfSe program was used to identify possible microbial biomarkers associated with PANS/PANDAS. As expected, the Bacteroidetes phylum was found to be a biomarker of y-PAN group, including Bacteroidaceae, Rikenellaceae, and Odoribacteriaceae at the family level as well as *Odoribacter*, *Bacteroides*, and *Oscillospira* at the genus level (**Figure 5A**). In particular, Bacteroidaceae were present in all y-PAN samples at higher levels than in the CTRL group (**Figure 5B**). On the other hand, the CTRL group GM was characterized by several Firmicutes family biomarkers, which were almost completely absent within y-PAN group. In addition, other biomarkers found in the CTRLs GM were absent or present at very low frequencies in the GM of PANS/PANDAS patients. Within Firmicutes phylum, potential biomarkers included Turicibacteriaceae, Erysipelotrichaceae, and Lachnospiraceae; the two latter were found at very low levels in y-PAN patients’ GM, with low distributions of the *Dorea*, *Roseburia*, and *Coprococcus* genus, while *Turicibacter* was absent.

**FIGURE 6:**
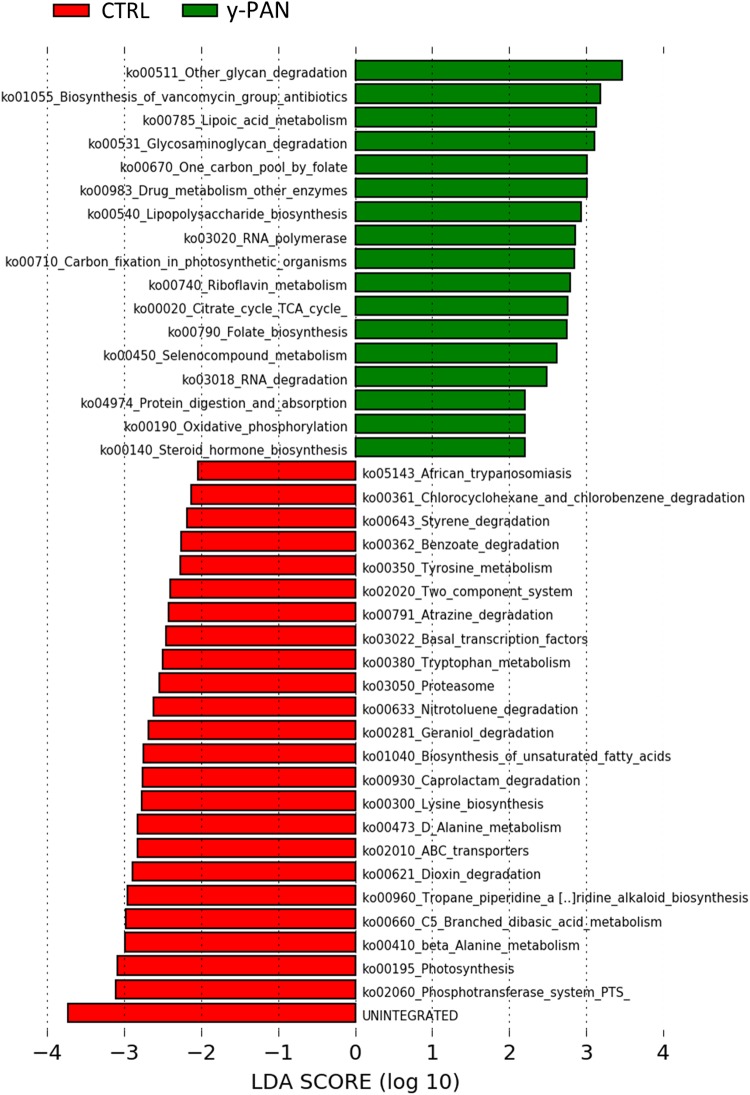
KEGG biomarkers associated with the y-PAN and CTRL groups. A linear discriminant effect size (LeFse) analysis has been performed (α value = 0.05, logarithmic LDA score threshold = 2.0).

By analyzing how these biomarkers correlated with the other OTUs within the microbiota ecology, a major cluster related to y-PAN patients was observed in which the three family biomarkers (i.e., Bacteroidaceae, Rikenellaceae, and Odoribacteriaceae) were located together with other microbial families (**Supplementary Figure S4**). This cluster in particular was also composed of other Bacteroidetes families (such as Barnesiellaceae and Prevotellaceae), Actinobacteria families (such as Coriobacteriaceae and Proponiobacteriaceae), Verrucomicrobiaceae, and an unknown family of Clostridiales. Within this cluster of families, which were all positively correlated with each other, Bacteroidaceae had the highest relative abundance and a strong negative correlation with all other cluster families. However, within the CTRL GM cluster, the identified OTUs exhibited a completely different ecology and correlation pattern. Indeed, multiple small clusters rather than one major one represented the microbial ecology. The particular GM composition of y-PAN patients was associated with a specific KEGG pathway (**Figure 6**), as highlighted by the PICRUSt analysis. Specifically, y-PAN microbiota expressed mostly KEGGs associated with glycan biosynthesis and degradation (i.e., ko00511, ko00531, and ko00540), fatty acids biosynthesis (ko00785), as well as energy (ko00020 and ko00450) and vitamin metabolism (ko00740, ko00790, and ko00670). In contrast, those KEGGs identified in the y-PAN group lacked several of the pathways expressed at high levels in the CTRL group, such as those involved in immune response modulation (ko00621 and ko01040) and in neurological functions (i.e., ko00350, ko00473, ko00300, ko00410, and ko00380).

### Microbiota Description in o-PAN Patients

The OTU distribution of the second group of o-PAN patients had a less organized profile. LEfSe analysis failed to identify specific microbial biomarkers associated with this group, while a simple Wilcoxon test detected four differences in family distributions between o-PAN and CTRL subjects (**Supplementary Figure S5**). In particular, the o-PAN group was characterized by higher levels of Peptostreptococcaceae and Erysipelotrichaceae and reduced levels of Rikenellaceae and Barnesiellaceae. Although these families showed significant differences with respect to the CTRL group, they did not show uniform distributions within o-PAN samples.

In general, o-PAN patients were characterized by a very high level of diversity as already highlighted by α-diversity analysis (**Figure 3B**), and therefore an uneven OTUs profile.

### Anti-streptolysin O Titer and Bacterial Genera Correlations

Pearson’s correlation was utilized to further investigate the possible relationship between PANS/PANDAS OTUs and ASLOT (**Table 1**). At first, this correlation was carried out using the entire dataset without any significant results. This analysis was repeated by selecting only ASLOT values higher than 500 units (Cardona and Orefici, 2001) and reported not more than 5 months prior to GM analysis to ensure titer stability (8/30 patients). The latter test showed a significant negative correlation between ASLOT and *Dehalobacterium*, *Corynebacterium*, *Gemella*, and *Lactobacillus* as well as a positive association with *Odoribacter* (**Supplementary Figure S6**).

## Discussion

Considering the distribution of α-diversity values calculated using the entire dataset of PANS/PANDAS patients, it was possible to observe a significant low value for both Observed and Chao1 indices, thus suggesting that a lower OTU number (including rare OTUs) distinguishes PANS/PANDAS patients from CTRL group. However, the Shannon index did not reveal a statistical significance between the two groups analyzed. The β-diversity analysis identified a clear cluster of CTRL subjects belonging to both the 4–6 and 7–8 age ranges that was substantially separate in composition, from a phylogenetic point of view (unweighted UniFrac), from the rest of the CTRL group subjects and from peer PANS/PANDAS patients. Indeed, age can greatly influence GM composition, especially during early stages of life (Rodríguez et al., 2015; Odamaki et al., 2016). Thus, on the basis of these data, PANS/PANDAS patients and the CTRL group subjects were split into two age groups [4–8 (y-PAN) and >9 years old (o-PAN)], to remove possible age-related bias in further analyses.

Re-performing α-diversity analyses on the two age groups, again a lower level of α-diversity was observed for PANS/PANDAS patients compared to CTRL subjects, confirming the data from the previous analysis. This seems to suggest a relationship between the disease and gut composition, regardless of the patients’ age range. The y-PAN patients had higher levels of homogeneity and very low standard deviations (i.e., Chao1 index), together with reduced levels of biodiversity compared to their control peers, showing an evident loss of species diversity in patients. Interestingly, concerning the Shannon index results, patients had an OTU distribution similar to that of the CTRL subjects. Thus, the loss of biodiversity was limited and not so evident in all α-diversity indices, possibly suggesting that y-PAN group is characterized by a low degree of dysbiosis, which is defined as an imbalanced OTU distribution within the GM, normally associated with some pathologic conditions (Carding et al., 2015). Therefore, it appears that the y-PAN microbiota is populated by fewer microbial species than CTRL GM, but in their own form of equilibrium within the intestinal tract.

At the phylum level, the Wilcoxon test highlighted the reduction of Firmicutes and TM7 phyla in y-PAN, along with a major enrichment in Bacteroidetes. This phylum was identified as a microbial marker by LEfSe analysis, thus suggesting that its abundance in the y-PAN gut is strongly inhibiting colonization by other phyla. Bacteroidetes is important to host energy yield through the fermentation of non-digestible polysaccharides and the production of short fatty acids (SCFAs) along with the CO_2_ and H_2_ gas_,_ which are consumed by other members of gut microbial community (Topping and Clifton, 2001; Macfarlane and Macfarlane, 2003; Johnson et al., 2017). Based on its metabolic functions, it is not surprising that Bacteroidetes plays such a central role in host physiology and that changes in its relative abundance are associated with metabolic disorders (Qin et al., 2012; Kolmeder et al., 2015).

Results obtained from y-PAN patients highlight the presence of a clear disequilibrium between Firmicutes and Bacteroidetes compared with subjects in the CTRL group; this disequilibrium may be responsible for altered metabolic function (Boulangé et al., 2016; Maier et al., 2017). Indeed, a different KEGG profile is found when comparing y-PAN and CTRL samples in the present study. Specifically, y-PAN samples were characterized by several signs of inflammation and increased cellular metabolism. In fact, their microbiota was enriched in glycan degradation, TCA cycle (tricarboxylic acid), and lipoic acid biosynthesis (a part of FA biosynthesis) capacities, which are part of a specific metabolic profile associated with a higher acetyl-CoA and SCFA production by gut microbial communities (Kim et al., 2016). Kim et al. (2016) have demonstrated that this same metabolic pathway leads to a higher B cell differentiation and Ig production, thus enhancing host antibody response. B cells play a primary role in immunity due to their antigen presentation capacity, which allows them to influence T cell differentiation (Courtemanche et al., 2004; Crawford et al., 2006). Moreover, y-PAN microbiota seems to have increased folate biosynthesis capacity that is known to influence the proliferation of CD8^+^ T cell lymphocytes (Huang et al., 1999). Given these data, it is possible to speculate that, in y-PAN subjects, the increase in this specific metabolic pathway in GM can lead to enhanced immune responses and perhaps even excess inflammation. This hypothesis is further supported by the presence in these patients of Odoribacteriaceae (genus *Odoribacter*), Bacteroidaceae (genus *Bacteroides*), and Rikenellaceae all of which are positively associated with pro-inflammatory status in several metabolic and autoimmune diseases (Swidsinski et al., 2005; Giannelli et al., 2014; Costello et al., 2015).

These three families, Odoribacteriaceae, Bacteroidaceae, and Rikenellaceae, are in the middle of a large bacterial cluster in the network analysis with strong positive or negative correlations. In particular, Bacteroidaceae, which is the most abundant family of the three biomarkers, exhibits a strong negative correlation to all other families; thus, its increase is paralleled by a disadvantage to all others.

An additional microbial biomarker found in y-PAN samples is *Oscillospira*, normally associated with healthy conditions (Zhu et al., 2013; Walters et al., 2014; Del Chierico et al., 2017). Its presence here is likely due to the higher abundance of *Bacteroides* in y-PAN patients; *Bacteroides* are strong producers of fermented products consumed by *Oscillospira* (Konikoff and Gophna, 2016).

However, the metabolic profile of y-PAN microbiota also characterized by the lack of several expected KEGGs founded in CTRL microbiota. First, there was a low presence of important anti-inflammatory elements such as dioxin degradation and unsaturated FA. While FA may inhibit the production of IL-1β, IL-6, IL-8, and TNFα, dioxin is a strong environment contaminant with immunomodulatory abilities due to its receptor (the aryl hydrocarbon receptor). Dioxin is found in all immune system cells and seems to play an important role in IBD (Arsenescu et al., 2011). The identification of these KEGG biomarkers in the CTRL group confirms the inflammatory status of y-PAN patients.

The present study gives evidence of the absence of specific pathways involved in certain neuronal functions, included a lower abundance microbiota capable of tyrosine metabolism in y-PAN patients. Tyrosine is a non-essential amino acid that is the major precursor of dopamine production via tyrosine hydroxylase (TH; Daubner et al., 2011). A reduction in tyrosine metabolism has been associated with some neuronal pathologies such as Parkinson’s disease (DiFrancisco-Donoghue et al., 2014). The link between tyrosine and dopamine metabolism could be an interesting perspective with which to interpret PANS and PANDAS disorder in young children, since this neurotransmitter regulates learning and modulates circadian rhythm (Korshunov et al., 2017). It has been proposed that the abnormalities in behaviors in y-PAN children may be caused by autoantibody activity against brain antigens (Cunningham and Cox, 2016), potentially induced by *Streptococcus pyogenes* infections through a process of cross-mimicry that leads to the production of antibodies against the dopamine receptor (Cox et al., 2013; Orefici et al., 2016). This same process may also shape the types of microbiota present in the gut by selecting for certain KEGG capacities in y-PAN patients. Modulation of dopamine production by the GM in PANS/PANDAS patients is further supported by the lack of Clostridiales families in y-PAN patients. Clostridia have a crucial role in gut homeostasis and host defense mechanisms against exogenous infections (Lopetuso et al., 2013). Interestingly, an *in vivo* experiment has found an association between the presence of Clostridia and increased norepinephrine and dopamine in the gut lumen (Asano et al., 2012). These data are in accordance with our finding of Clostridia almost exclusively in the CTRL group compared to the PANS/PANDAS group.

Interestingly, CTRL subjects exhibited higher levels of *Roseburia* genus members (Lachnospiraceae, Clostridial cluster XIVa); this observation deserves attention as this genus contributes to the maintenance of gut homeostasis, the ability to preserve gut barrier function, and for its anti-inflammatory effect due to butyrate production (Velasquez-Manoff, 2015).

In addition, D-alanine metabolism, as a KEGG biomarker, was increased in samples from CTRL subjects. D-Alanine is a D-amino acid present in the human CNS and is a strong agonist at the *N*-methyl-D-aspartate (NMDA)–glycine binding site (McBain et al., 1989). Hypofunction of D-alanine and NMDA receptors has been associated with some neurological dysfunctions such as encephalitis and schizophrenia (Tsai et al., 2006). Recently, antibodies against NMDA receptors have been detected after viral infection (Gable et al., 2009). Given these data, it is possible to speculate that the low level of D-alanine in y-PAN patients is due to an unknown antibody-mediated process linked to the streptococcal infection. During infection, anti-streptococcal/anti-neuronal antibodies are generated. It is possible that they are able to mediate the destruction of healthy neurons (via the NMDA receptor) through a mechanism of molecular mimics (Murphy and Pichichero, 2002).

Gut microbiota from the y-PAN patients was also characterized by the absence of the Erysipelotrichaceae family, which seems to be correlated with several immunologic conditions. Erysipelotrichaceae species may induce different immunologic responses; for example, Erysipelotrichaceae have been found to attract more IgA molecules than other gut microbial families (Palm et al., 2014). When trying to understand their absence in y-PAN patients’ GM, it is interesting to note that y-PAN patients may also have an IgA deficiency (Kawikova et al., 2010).

In the second group of o-PAN (≥9 years old) patients, Erysipelotrichaceae were in some way more abundant in the GM than in CTRL GM, although the great variability within the o-PAN group must be underlined. Indeed, LEfSe analysis failed in this case to find a pathological biomarker of this great variability in OTU profiles within the o-PAN group. The high heterogeneity within this group of patients is probably linked to the intense and repeated antibiotic treatments that they have undergone during their life. Indeed, antibiotic treatment seriously affects microbiota composition and function, sometimes even producing long-term deleterious effects (Becattini et al., 2016). Therefore, repeated antibiotic therapy (in association with other pharmacological treatments) has probably altered GM composition, making it difficult to detect possible microbial markers associated with the o-PAN syndrome. Thus, the GM in y-PAN subjects can be regarded as being in a “naïve” and in a baseline state from pathophysiological point of view. Future studies should better elucidate how microbiota changes during early stages of y-PAN disease in order to clarify the confounding effects of antibiotic therapy.

A Pearson’s test indicated that ASLOT is correlated with several microbial genera that are present in a subset of PANS/PANDAS patients characterized by anti-streptolysin O values higher than 500 units. Several negative correlations were obtained with a genus belonging to the Firmicutes phylum, which seems to be in accordance with the low percentage of this phylum among naïve y-PAN patients. On the other hand, *Odoribacter* were strongly positively correlated with ASLOT values, which is very interesting given that the same genus was also identified as a putative microbial biomarker from the LEfSe analysis in y-PAN patients.

Other cases have been reported in which *Odoribacter* seems to correlate with high inflammation levels and with behavioral dysfunction (including Alzheimer’s disease and autism) (Bajaj et al., 2012; De Angelis et al., 2015; Shen et al., 2017). A recent study has demonstrated that bacterial strains involved in periodontitis (such as *Porphyromonas gingivalis*) lead to systemic inflammation, even in distant body sites such as GM via swallowing (Hajishengallis, 2014). Therefore, given the involvement of *S. pyogenes* infection in PANS/PANDAS etiology, it is plausible that the infection influences GM composition and host inflammation by the same mechanism.

## Conclusion

**FIGURE 7:**
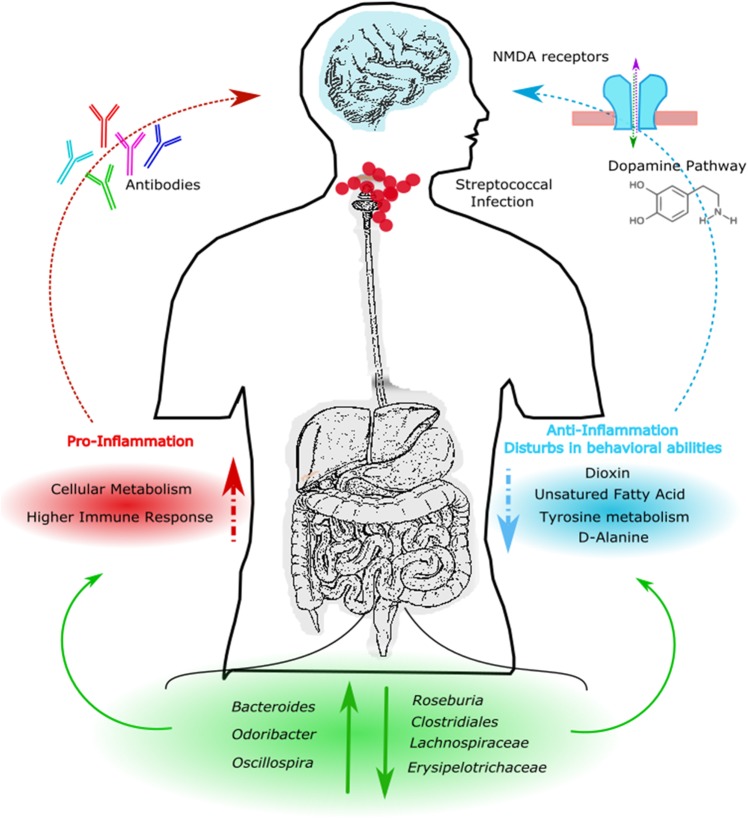
Descriptive model of PANS/PANDAS brain–gut microbiota axis. Model suggests that streptococcal infection can alter gut microbial communities leading to an increment of *Bacteroides*, *Odoribacter*, and *Oscillospira*, and to a reduction of *Roseburia*, Clostridiales, Lachnospiraceae, and Erysipelotrichaceae. KEGG analysis showed an increment of pro-inflammatory pathways and a reduction of anti-inflammatory and neurological predicted metabolites, respectively. This condition could affect dopamine pathways, *N*-methyl-D-aspartate (NMDA)–glycine binding site and antibodies modulation leading to behavior impairments.

The results obtained from this study suggest that streptococcal infections can alter gut microbial communities leading to a pro-inflammatory state in the gut by selecting for specific bacterial strains that are normally associated with gut inflammation and activation of the immune response (**Figure 7**). This condition is likely maintained in patients, even after the infection itself has resolved. Moreover, an altered GM composition could have indirect effects by reducing the production of metabolites involved in important brain functions such as SCFA, D-alanine and tyrosine metabolism, and the dopamine pathway. Thus, the GM composition may possibly influence behavior, as clinically observed in PANS/PANDAS patients.

## Author Contributions

AQ: data analysis and interpretation and manuscript writing. FDC: healthy control enrollment, sample collection, and manuscript revision. AR: data acquisition. SR: sample collection and data acquisition. GC: patient recruitment. LL: patient recruitment, clinical data collection, and manuscript revision. GI: patient recruitment and clinical data collection. BD: manuscript revision. FC: study conception and design. AG: study conception and design and manuscript revision. LP: study conception and design and manuscript writing.

## Conflict of Interest Statement

The authors declare that the research was conducted in the absence of any commercial or financial relationships that could be construed as a potential conflict of interest.

## Footnotes

1 http://www.ncbi.nlm.nih.gov/bioproject/?term=

2 http://www.micca.org/

3 http://huttenhower.sph.harvard.edu/humann2

## Supplementary Material

FIGURE S1 Sample selections based on the α-diversity analysis. The Observed index was used to evaluate sample biodiversity level for each patient. In this plot, patient ID is reported on the *x*-axis while the number of reads is reported on the *y*-axis. Sphere dimension indicates α-diversity index values, while color intensity reads values, respectively.

FIGURE S2 Bar chart representing mean relative abundance of operational taxonomic units (OTUs) at the family and genus level. The bar plots represent the mean relative abundance of the 20 most abundant taxa at family **(a)** and genus **(b)** level for CTRL, o-PAN, and y-PAN groups.

FIGURE S3 Bar charts representing Wilcoxon test results on operational taxonomic units (OTUs) at the family level of the CTRL and y-PAN groups. Bars for y-PAN patients are reported in blue. Bars are reported in orange for CTRL. Only statistically significant comparisons are reported in the figure (*p* < 0.05).

FIGURE S4 Network analysis. Spearman’s correlation analysis among OTUs between the CTRL **(a)** and y-PAN **(b)** groups. Negative correlations are indicated by red lines. Positive correlations are indicated by blue lines.

FIGURE S5 Bar chart representing Wilcoxon test results on operational taxonomic units (OTUs) at the family level between the CTRL and o-PAN groups. Bars for o-PAN patients are reported in blue, while orange bars indicate CTRL patients. Only statistically significant comparisons are reported in the figure (*p* < 0.05).

FIGURE S6 Pearson’s correlation test between ASLOT values and microbial genus in PANS/PANDAS patients. Patients (8/30) were selected basing on ASLOT values >500 units and time until detection <5 months from GM analysis. Both negative and positive correlations (red and blue arrows, respectively) are considered significant at *p* < 0.05. Rho values are reported for each correlation (*r*).
