# Supplementary figures and images for: Gut Microbiota Profiling and Gut–Brain Crosstalk in Children Affected by Pediatric Acute-Onset Neuropsychiatric Syndrome and Pediatric Autoimmune Neuropsychiatric Disorders Associated With Streptococcal Infections
Source: Front Microbiol. 2018 Apr 6;9:675. doi: 10.3389/fmicb.2018.00675 (PMC5900790; doi:10.3389/fmicb.2018.00675)

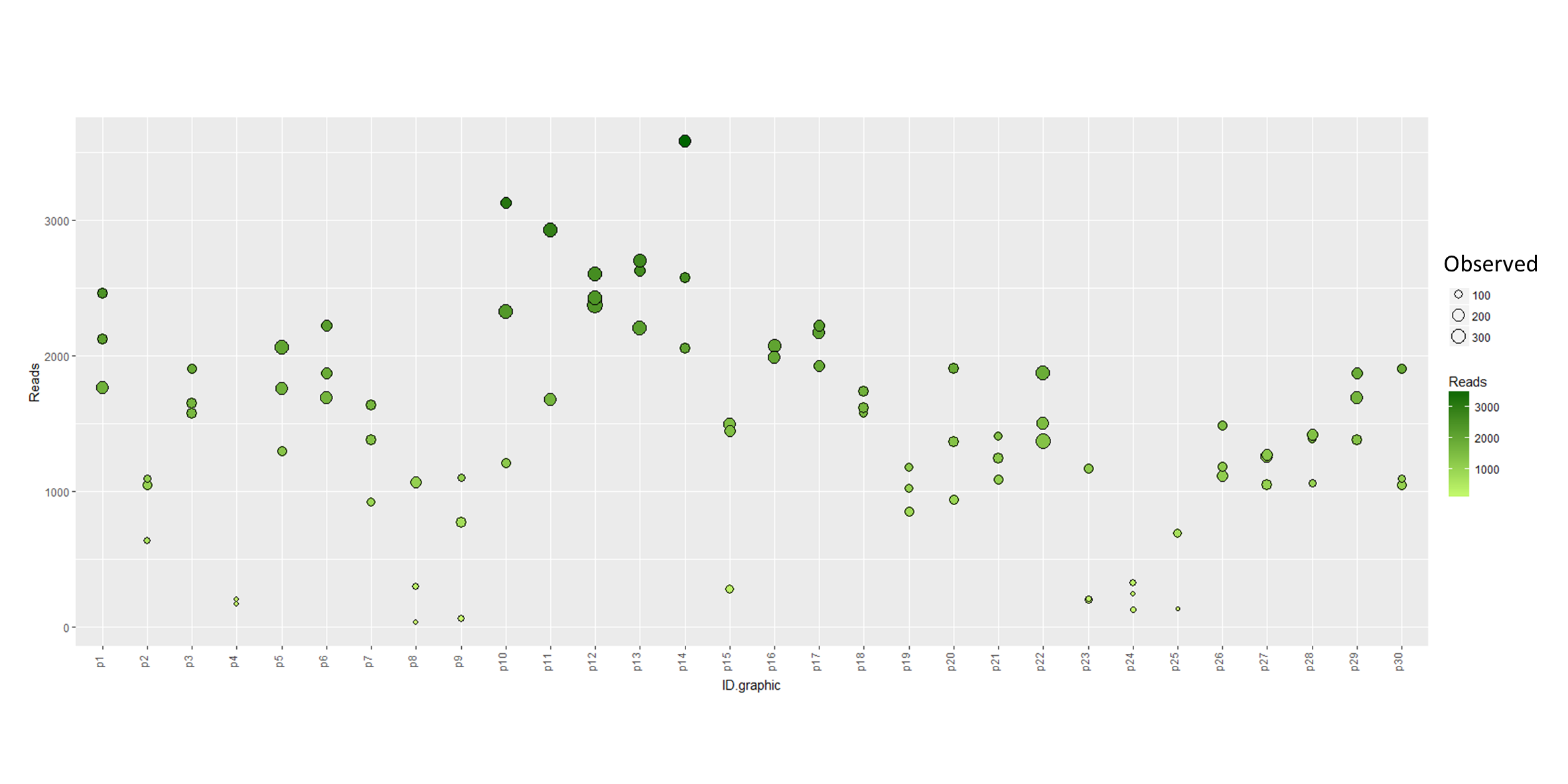

Supplement: FIGURE S1 — Sample selections based on the α-diversity analysis. The Observed index was used to evaluate sample biodiversity level for each patient. In this plot, patient ID is reported on the x-axis while the number of reads is reported on the y-axis. Sphere dimension indicates α-diversity index values, while color intensity reads values, respectively. [file Image_1.TIF]

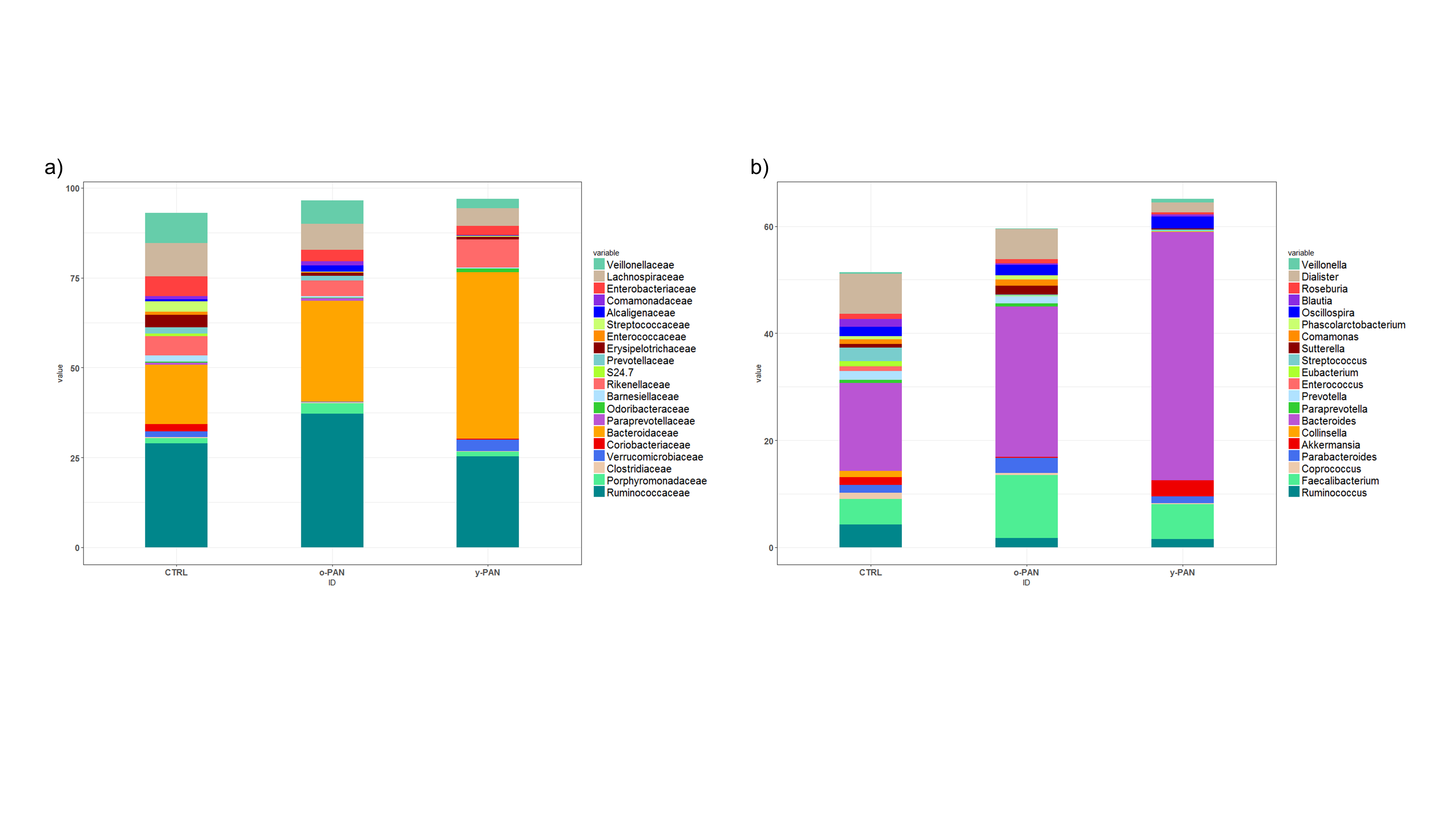

Supplement: FIGURE S2 — Bar chart representing mean relative abundance of operational taxonomic units (OTUs) at the family and genus level. The bar plots represent the mean relative abundance of the 20 most abundant taxa at family (a) and genus (b) level for CTRL, o-PAN, and y-PAN groups. [file Image_2.TIF]

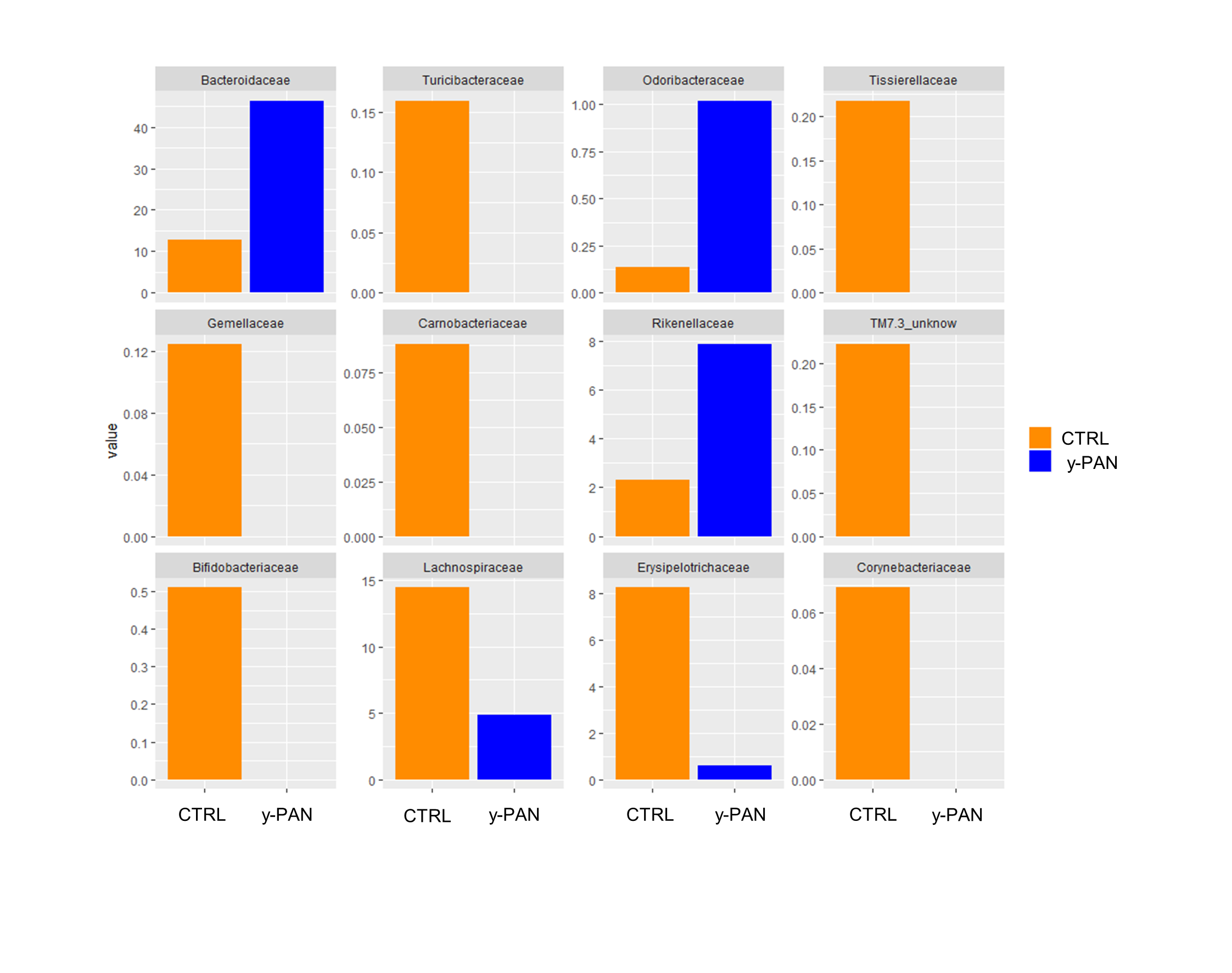

Supplement: FIGURE S3 — Bar charts representing Wilcoxon test results on operational taxonomic units (OTUs) at the family level of the CTRL and y-PAN groups. Bars for y-PAN patients are reported in blue. Bars are reported in orange for CTRL. Only statistically significant comparisons are reported in the figure (p < 0.05). [file Image_3.TIF]

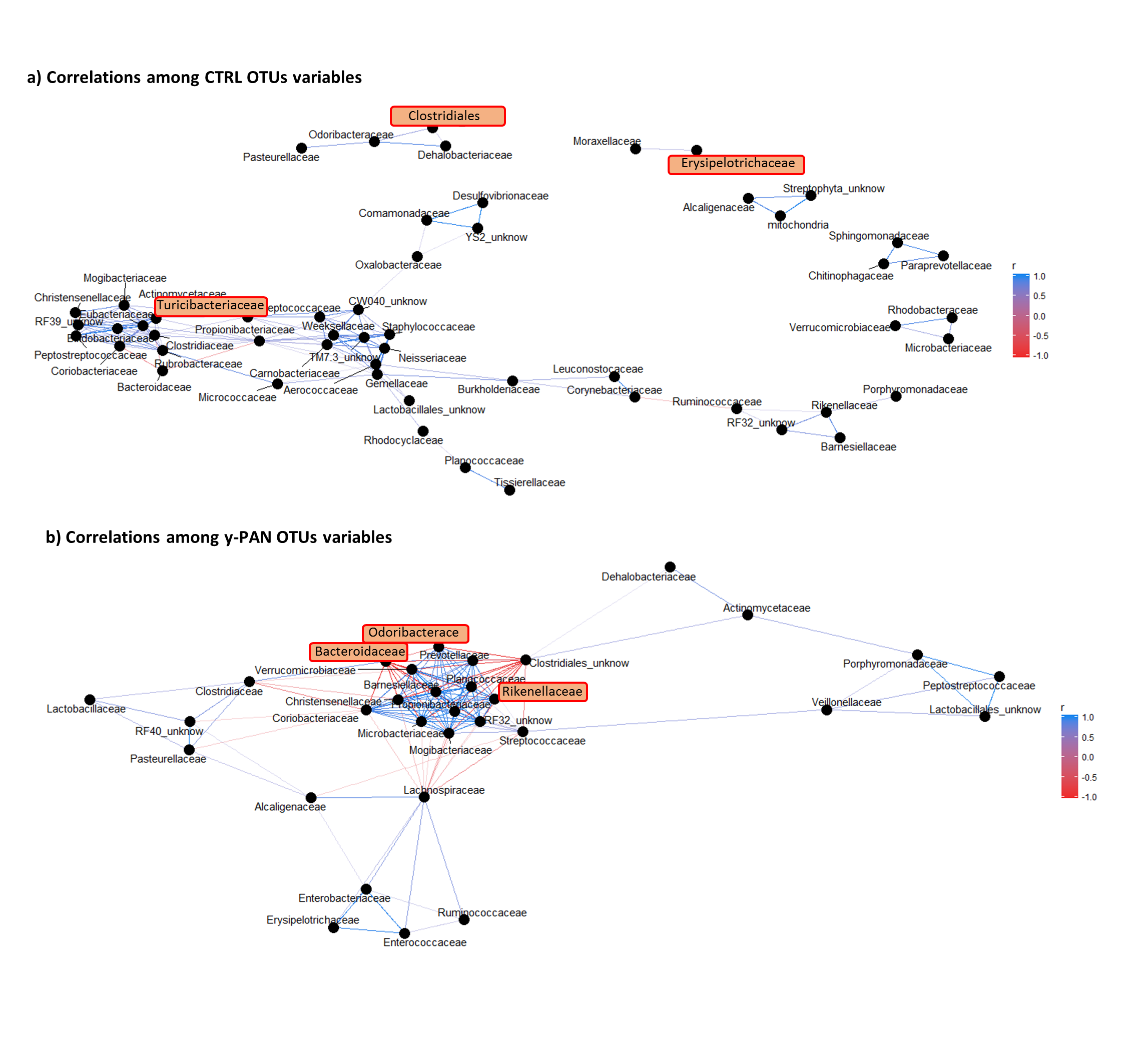

Supplement: FIGURE S4 — Network analysis. Spearman’s correlation analysis among OTUs between the CTRL (a) and y-PAN (b) groups. Negative correlations are indicated by red lines. Positive correlations are indicated by blue lines. [file Image_4.TIF]

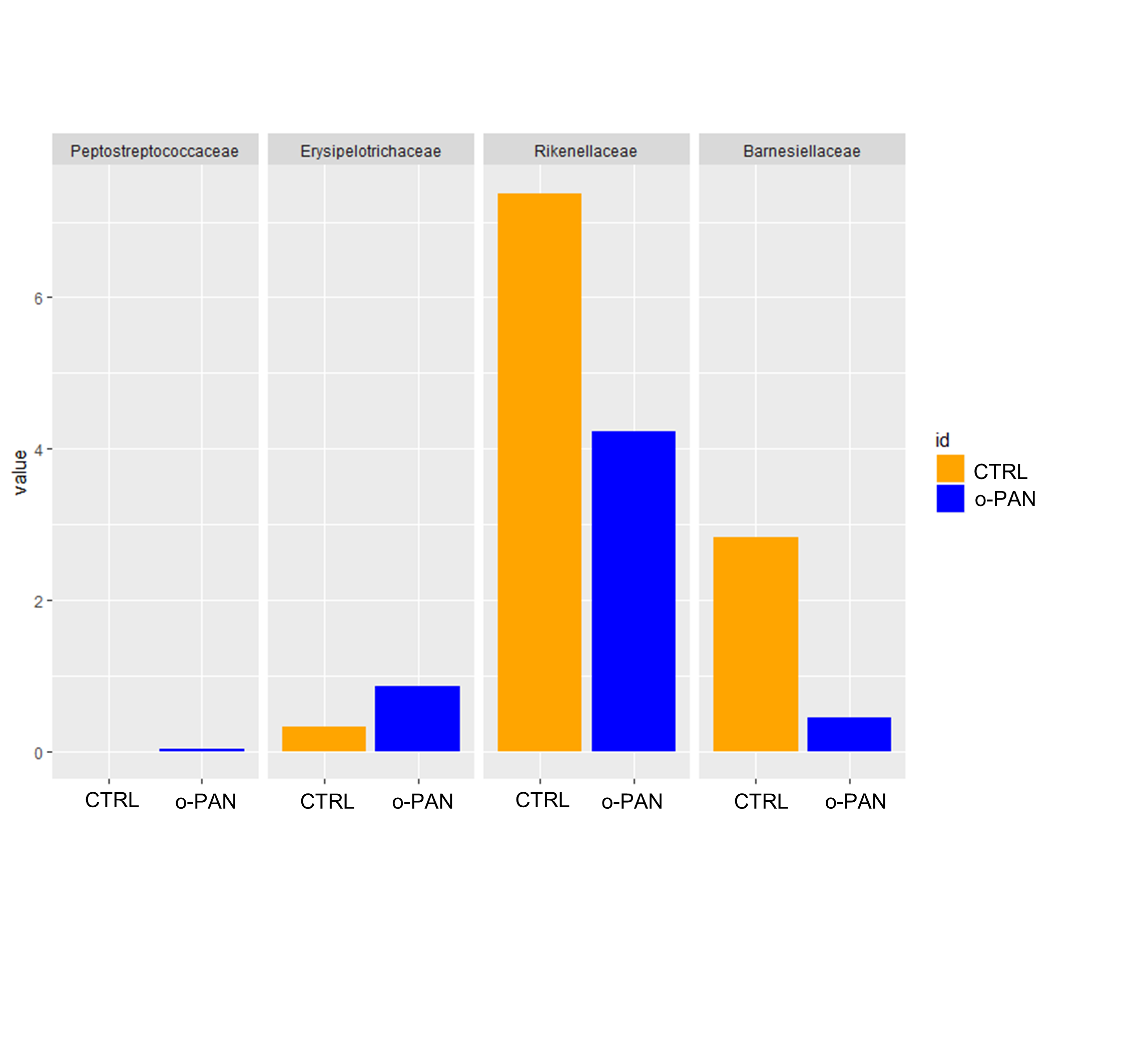

Supplement: FIGURE S5 — Bar chart representing Wilcoxon test results on operational taxonomic units (OTUs) at the family level between the CTRL and o-PAN groups. Bars for o-PAN patients are reported in blue, while orange bars indicate CTRL patients. Only statistically significant comparisons are reported in the figure (p < 0.05). [file Image_5.TIF]

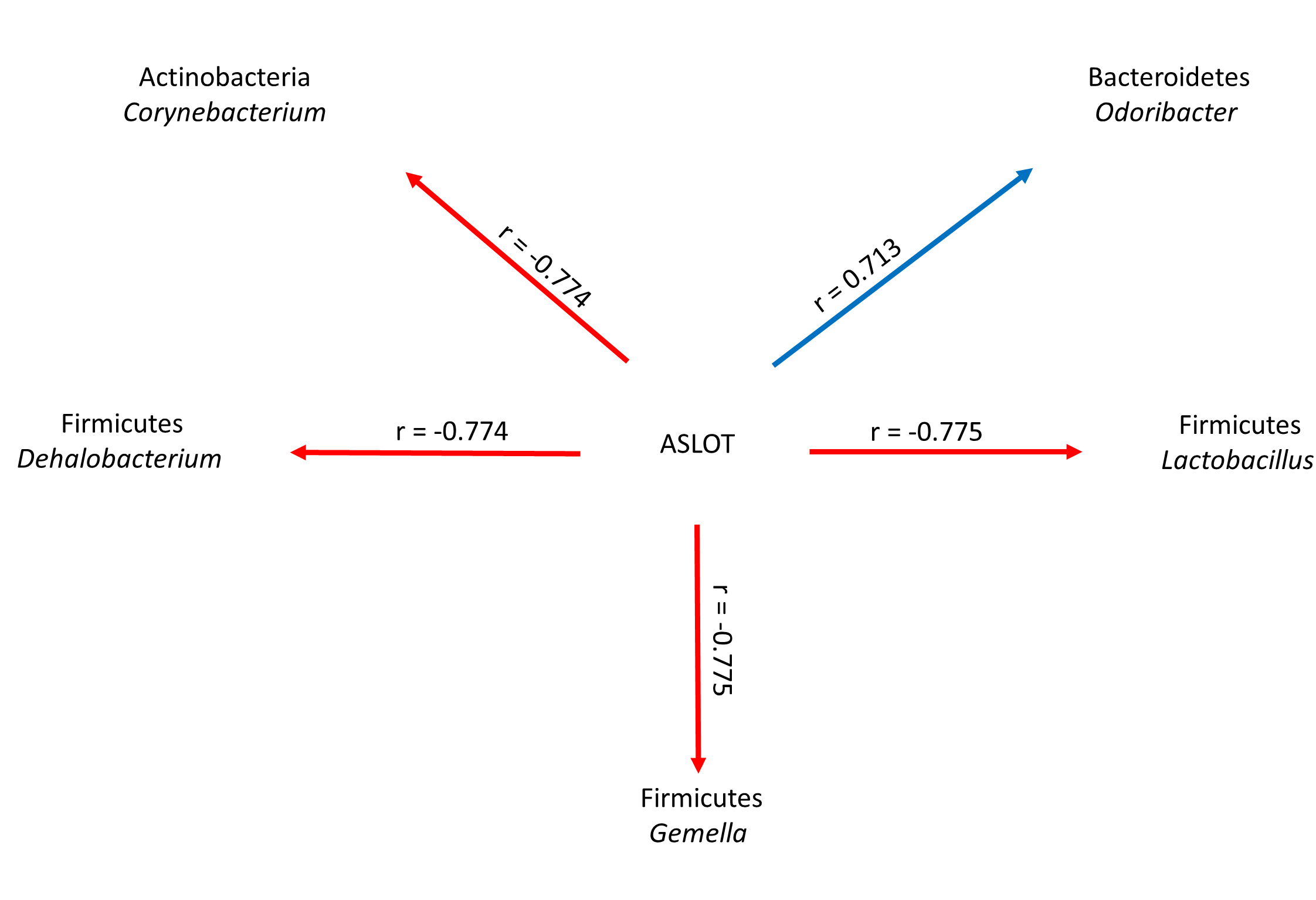

Supplement: FIGURE S6 — Pearson’s correlation test between ASLOT values and microbial genus in PANS/PANDAS patients. Patients (8/30) were selected basing on ASLOT values >500 units and time until detection <5 months from GM analysis. Both negative and positive correlations (red and blue arrows, respectively) are considered significant at p < 0.05. Rho values are reported for each correlation (r). [file Image_6.TIF]
